# Melatonin Therapy Prevents Programmed Hypertension and Nitric Oxide Deficiency in Offspring Exposed to Maternal Caloric Restriction

**DOI:** 10.1155/2014/283180

**Published:** 2014-04-22

**Authors:** You-Lin Tain, Li-Tung Huang, Chien-Ning Hsu, Chien-Te Lee

**Affiliations:** ^1^Department of Pediatrics, Kaohsiung Chang Gung Memorial Hospital and College of Medicine, Chang Gung University, Kaohsiung 833, Taiwan; ^2^Center for Translational Research in Biomedical Sciences, Kaohsiung Chang Gung Memorial Hospital and College of Medicine, Chang Gung University, Kaohsiung 833, Taiwan; ^3^Department of Traditional Chinese Medicine, Chang Gung University, Linkou 333, Taiwan; ^4^Department of Pharmacy, Kaohsiung Chang Gung Memorial Hospital and College of Medicine, Chang Gung University, Kaohsiung 833, Taiwan; ^5^Graduate Institute of Clinical Pharmacy, College of Pharmacy, Kaohsiung Medical University, Kaohsiung 833, Taiwan; ^6^Division of Nephrology, Department of Internal Medicine, Kaohsiung Chang Gung Memorial Hospital and College of Medicine, Chang Gung University, Kaohsiung 833, Taiwan

## Abstract

Nitric oxide (NO) deficiency is involved in the development of hypertension, a condition that can originate early in life. We examined whether NO deficiency contributed to programmed hypertension in offspring from mothers with calorie-restricted diets and whether melatonin therapy prevented this process. We examined 3-month-old male rat offspring from four maternal groups: untreated controls, 50% calorie-restricted (CR) rats, controls treated with melatonin (0.01% in drinking water), and CR rats treated with melatonin (CR + M). The effect of melatonin on nephrogenesis was analyzed using next-generation sequencing. The CR group developed hypertension associated with elevated plasma asymmetric dimethylarginine (ADMA, a nitric oxide synthase inhibitor), decreased L-arginine, decreased L-arginine-to-ADMA ratio (AAR), and decreased renal NO production. Maternal melatonin treatment prevented these effects. Melatonin prevented CR-induced renin and prorenin receptor expression. Renal angiotensin-converting enzyme 2 protein levels in the M and CR + M groups were also significantly increased by melatonin therapy. Maternal melatonin therapy had long-term epigenetic effects on global gene expression in the kidneys of offspring. Conclusively, we attributed these protective effects of melatonin on CR-induced programmed hypertension to the reduction of plasma ADMA, restoration of plasma AAR, increase of renal NO level, alteration of renin-angiotensin system, and epigenetic changes in numerous genes.

## 1. Introduction

Hypertension might originate during early life. Maternal malnutrition can impair development, resulting in intrauterine growth restriction (IUGR), permanent structural changes, and disrupted physiological function—a phenomenon called “developmental programming” [1]. In the kidneys of both humans and experimental models, developmental programming reduces nephron numbers, alters the renin-angiotensin system (RAS), and impairs natriuresis, leading to adult kidney disease and hypertension [2–5].

A number of hypotheses have been proposed to explain the developmental programming phenomenon [6]. Oxidative stress is proposed as the underlying link between developmental programing and elevated risks of hypertension and kidney disease in adulthood [7, 8]. Asymmetric dimethylarginine (ADMA), an endogenous inhibitor of NO synthase (NOS), causes oxidative stress and is involved in the development of hypertension [9]. Our recent work demonstrated that an impaired ADMA-NO pathway and low nephron numbers are associated with programmed hypertension in the adult offspring of malnourished or diabetic mothers [10, 11]. Reduced nephron numbers impaired renal tubular sodium reabsorption, and the altered RAS components disrupted sodium retention, ultimately increasing blood pressure (BP) and inducing kidney damage. Histone deacetylases (HDACs) repress gene expression, a mechanism of epigenetic control that is involved in developmental programming. Class I HDACs are critical in nephrogenesis, particularly HDAC1-3 that are highly expressed in nephron precursors [12]. HDACs also play an important role in regulating RAS components during nephrogenesis [13]. These observations suggest that these mechanisms jointly lead to the development of hypertension and kidney disease.

Melatonin, an indoleamine produced from the pineal gland, is an antioxidant and free radical scavenger [14]. Experimental and human studies indicate that melatonin can regulate BP [10, 11]. We recently found that melatonin can prevent oxidative stress and hypertension concurrently in young spontaneously hypertensive rats (SHR) [15]. Emerging evidence supports novel roles of melatonin in epigenetic modulation through the regulation of HDACs [16, 17]. Thus, we examined whether melatonin prevented programmed hypertension in offspring exposed to maternal caloric restriction through reduction of oxidative stress, alteration of the RAS pathway, and modulation of HDACs. Moreover, we identified melatonin-induced gene changes during nephrogenesis and determined whether melatonin treatment induced global changes in biological processes by using next-generation sequencing.

## 2. Material and Methods

### 2.1. Animal Models

This study was carried out in strict accordance with the recommendations of the Guide for the Care and Use of Laboratory Animals of the National Institutes of Health. The protocol was approved by the Institutional Animal Care and Use Committee of the Kaohsiung Chang Gung Memorial Hospital. Virgin Sprague-Dawley (SD) rats (12–16 weeks old) were obtained from BioLASCO Taiwan Co., Ltd. (Taipei, Taiwan), and were housed and maintained in a facility accredited by the Association for Assessment and Accreditation of Laboratory Animal Care International. Male SD rats were caged with individual females until mating was confirmed. Calorie-restricted (CR) maternal rats received 11 g/d of a standard chow from day 11 of pregnancy until the day of delivery (day 23) and 20 g/d during the entire lactation period [10]. A subset of CR mothers was treated for the duration of the pregnancy with 0.01% melatonin dissolved in drinking water (CR + M, *n* = 8). The control group (*n* = 8) mothers had free access to standard rat chow. As another control, maternal rats were allowed free access to standard rat chow and were treated with 0.01% melatonin in drinking water (M, *n* = 10). After birth, each litter was left with the mother until weaning; pups were not weighed at birth to prevent maternal rejection. Male offspring, selected at random from each litter, were used in all subsequent experiments. In rats, nephrogenesis occurs predominantly from late gestation to 1-2 weeks postnatum and litters were typically weaned by postnatal week 3. Thus, melatonin was administered to mother rats for a total period of 6 weeks to cover the entire period of nephrogenesis. The dose of melatonin used was based on our previous study [15]. Water bottles were covered with aluminum foil to protect them from light. BP was measured in conscious rats by using an indirect tail-cuff method (BP-2000, Visitech Systems, Inc., Apex, NC, USA) after they had been systematically trained [10]. Three stable consecutive measures were taken and averaged. All offspring were sacrificed at 12 weeks of age and heparinized blood samples were collected. Kidneys were harvested after perfusion with PBS, divided into cortex and medulla regions, and snap-frozen. The activity of dimethylarginine dimethylaminohydrolase (DDAH), an ADMA-metabolizing enzyme, was measured using a colorimetric assay. The assay determined the rate of l-citrulline production and we performed the assay as previously described [18].

### 2.2. High-Performance Liquid Chromatography (HPLC)

Plasma and kidney l-arginine, l-citrulline, ADMA, and symmetric dimethylarginine (SDMA, a stereoisomer of ADMA) levels were measured using HPLC (HP series 1100, Agilent Technologies, Inc., Santa Clara, CA, USA) with the OPA-3MPA derivatization reagent as we described previously [10]. Standards contained l-arginine, l-citrulline, ADMA, and SDMA in the range of 1–100 *μ*M, 1–100 *μ*M, 0.5–5 *μ*M, and 0.5–5 *μ*M, respectively. The recovery rate was between 90 and 105%. The tissue concentration was factored for protein concentration, which was represented as *μ*mol/mg protein. Plasma and urine creatinine (Cr) levels were analyzed by HPLC as described previously [10]. The creatinine clearance (CCr) was calculated by dividing the total amount of Cr excreted in urine by the Cr concentration in plasma. CCr values were normalized with respect to body weight.

### 2.3. Electron Paramagnetic Resonance (EPR)

Superoxide production was measured by EPR spectroscopy using a 1-hydroxy-3-carboxypyrrolidine (CPH) hydroxylamine spin probe, as we previously described [11]. The EPR spectra were recorded using an EMX Plus EPR spectrometer (Bruker BioSpin, Rheinstetten, Germany) equipped with an EMX-m40X microwave bridge operating at 9.87 GHz. NO was detected by EPR using N-methyl-D-glucamine dithiocarbamate (MGD) spin probe and FeSO_4_, as previously described [11]. The EPR spectra were recorded using an EMX Plus EPR spectrometer (Bruker BioSpin) equipped with an EMX-m40X microwave bridge operating at 3.16 GHz.

### 2.4. Metanephros Organ Culture

Metanephros organ culture was performed as we described previously [11]. Briefly, SD female rats of known mating date were anesthetized and laparotomized. Fetuses were aseptically removed, and metanephroi from fetuses at embryonic day 14 (E14) were collected and freed of exogenous tissue. Explants were placed onto a Steritop filter unit (Millipore, Billerica, MA, USA) floating on a defined serum-free medium and incubated for 6 d in 35 mm Petri dishes at 37°C in a humidified incubator (5% CO_2_). The defined medium was composed of Eagle's Minimum Essential Medium containing 10% (v/v) fetal calf serum, 100 units/mL penicillin, and 100 *μ*g/mL streptomycin. All of these reagents were obtained from Sigma (St. Louis, MO, USA). The culture medium was changed daily, and no antibiotic or fungicide was present throughout the experiment. Fresh aliquots of each culture medium additive were used for each metanephros culture. The medium was changed daily. Metanephroi were treated with melatonin (1 *μ*M and 1 mM) and harvested after 6 d for real-time polymerase chain reaction.

### 2.5. Quantitative Real-Time Polymerase Chain Reaction (PCR)

RNA was extracted as described previously [10]. Two-step quantitative real-time PCR was conducted using the QuantiTect SYBR Green PCR Kit (Qiagen, Valencia, CA, USA) and the iCycler iQ Multicolor Real-Time PCR Detection System (Bio-Rad, Hercules, CA, USA). Nephron deficit was assessed by changes in the expression factors known to be involved in branching morphogenesis (BMP4, FGF2, and PAX2) and apoptosis (p53 and Bax). Several components of the RAS were analyzed including renin, prorenin receptor (PRR), angiotensinogen (AGT), angiotensin-converting enzyme (ACE), ACE2, angiotensin II type 1 (AT1R) and 2 receptor (AT2R), and angiotensin (1–7) MAS receptor. Class I HDACs, HDAC-1, -2, -3, and -8, were also examined. We used 18S rRNA (r18S) as a reference. Primers were designed using GeneTool Software (BioTools, Edmonton, Alberta, Canada) (Table 1). All samples were run in duplicate. To quantify the relative gene expression, the comparative threshold cycle (C_T_) method was employed. For each sample, the average C_T_ value was subtracted from the corresponding average r18S value, calculating the ΔC_T_. ΔΔC_T_ was calculated by subtracting the average control ΔC_T_ value from the average experimental ΔC_T_. The fold-increase of the experimental sample relative to the control was calculated using the formula 2^−ΔΔC_T_^.

### 2.6. Western Blot

Western blot analysis was performed as previously described [10]. We used the following antibodies from Santa Cruz Biotechnology (Santa Cruz, CA, USA): rabbit polyclonal anti-rat PRR (1 : 500, overnight incubation), rabbit anti-rat ACE2 (1 : 1000, overnight incubation), rabbit anti-rat AT1R (1 : 250, overnight incubation), rabbit anti-rat AT2R (1 : 250, overnight incubation), and rabbit anti-rat MAS (1 : 1000, overnight incubation; Santa Cruz Biotechnology). The bands of interest were visualized using enhanced chemiluminescence reagent (PerkinElmer, Waltham, MA, USA) and quantified by densitometry (Quantity One Analysis software, Bio-Rad). Band density was calculated as the integrated optical density (IOD) minus the background value. The density of Ponceau red staining (PonS) was used to correct for variations in total protein loading. Protein abundance was calculated as IOD/PonS.

### 2.7. Next-Generation Sequencing and Analysis

In rats, nephrogenesis occurs predominantly from late gestation to 7–10 days postnatum. Thus, offspring from the control and M groups were sacrificed at 1 week of age. Kidneys were isolated and snap-frozen for whole-genome RNA next-generation sequencing (RNA-seq), performed by Welgene Biotech Co., Ltd. (Taipei, Taiwan). Purified RNA was quantified at 260 nm (OD_600_) by using ND-1000 spectrophotometer (Nanodrop Technology, Wilmington, DE, USA) and analyzed using a Bioanalyzer 2100 (Agilent Technology) with RNA 6000 LabChip kit (Agilent Technologies). All procedures were performed according to the Illumina protocol. For all samples, library construction was performed using the TruSeq RNA Sample Prep Kit v2 for ∼160 bp (single-end) sequencing and the Solexa platform. The sequence was directly determined by sequencing-by-synthesis technology using the TruSeq SBS Kit. Raw sequences were obtained using the Illumina GA Pipeline software CASAVA v1.8, which was expected to generate 10 million reads per sample. Quantification for gene expression was calculated as reads per kilobase of exon per million mapped reads [19]. For differential expression analysis, Cufflink v 2.1.1 and CummeRbund v 2.0.0 were used to perform statistical analyses of the gene expression profiles. The reference genome and gene annotations were retrieved from the Ensembl database (http://asia.ensembl.org/index.html). Gene ontology analysis for significant genes was performed using KEGG (http://www.genome.jp/kegg/) and NIH DAVID Bioinformatics Resources 6.7 (http://david.abcc.ncifcrf.gov/) to identify regulated biological themes.

### 2.8. Statistical Analysis

TheShapiro-Wilk normality test was used to determine which data were normally distributed. Normally distributed data are given as mean ± standard error of the mean. For most parameters, statistical analysis was performed using one-way analysis of variance (ANOVA) and Tukey's post hoc test for multiple comparisons. BP was analyzed by two-way repeated-measures ANOVA and Tukey's post hoc test. A *P* value < 0.05 was considered statistically significant. Analyses were performed using the Statistical Package for the Social Sciences (SPSS) software (Chicago, IL, USA).

## 3. Results

### 3.1. The Effects of Melatonin on Morphological and Biochemical Values in CR Rats

**Figure 1:**
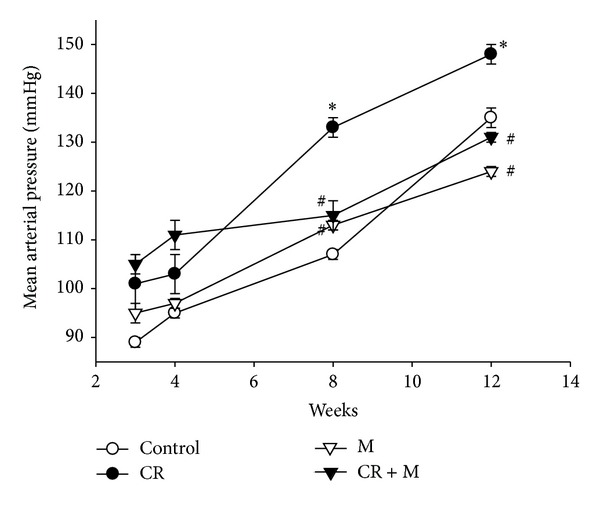
Effect of melatonin and caloric restriction (CR) on mean arterial pressure in male offspring at 12 weeks of age.  *P < 0.05 versus control; ^#^ *P* < 0.05 versus CR.

Litter sizes were not significantly altered by caloric restriction in the mother rat or by melatonin treatment. The amounts of water intake and urine output were not significantly different in the control and CR groups. Male pup mortality rates did not differ between the four groups analyzed. As shown in Table 2, the CR and M groups had lower and higher body weight (BW) than the control at 12 weeks of age, respectively, whereas the CR + M group had an intermediate BW. Kidney weight and kidney weight-to-BW ratio did not differ between the control and CR groups. Melatonin significantly increased kidney weight and kidney weight-to-BW ratio in the M and CR + M groups. Although heart weight was not different between control and CR groups, the heart weight-to-BW ratio was greater in the CR group. Melatonin caused increased heart weight and heart weight-to-BW ratio in the M group, but not in the CR + M group. CR increased systolic and diastolic BP and mean arterial pressure at 12 weeks of age. Melatonin therapy prevented these effects of CR. In addition, melatonin therapy reduced diastolic BP and mean arterial pressure in the M group compared to the control. As shown in Figure 1, mean arterial pressure was similar in the four groups at 4 weeks of age. By 8 weeks of age, mean arterial pressure had increased in the CR group relative to controls. A significant reduction in mean arterial pressure was measured in the M and CR + M groups versus the control at 8 and 12 weeks of age. In contrast, plasma creatinine level did not differ between the four groups. These data demonstrated that CR induced programmed hypertension but had no effect on renal function on 12-week-old offspring.

### 3.2. The Effects of Melatonin on l-Arginine, l-Citrulline, and Dimethylarginine Levels

As shown in Table 3, plasma levels of ADMA and SDMA were elevated nearly 70% and 150% following maternal CR, respectively. In contrast, the l-arginine levels and l-arginine-to-ADMA ratio were decreased by 30% and 55%, respectively. Melatonin treatment significantly increased l-arginine levels and l-arginine-to-ADMA ratio, but decreased ADMA and SDMA levels in the CR + M group. In the kidney, levels of l-citrulline, l-arginine, ADMA, and SDMA did not differ between the four groups. However, renal l-arginine-to-ADMA ratio was higher in the CR + M group versus the M group. We next analyzed superoxide and NO production in the kidney by using EPR. We found no difference in renal superoxide level among the four groups (control: 745 ± 28, CR: 823 ± 107, M: 665 ± 35, CR + M: 757 ± 42 arbitrary units; *P* > 0.05). CR significantly reduced renal NO levels, but not in the presence of melatonin (control: 412 ± 43, CR: 284 ± 18, M: 308 ± 34, CR + M: 414 ± 55 arbitrary units; control versus CR, *P* < 0.05; CR versus CR + M, *P* < 0.05).

### 3.3. The Effects of Melatonin on the ADMA Pathway

**Figure 2:**
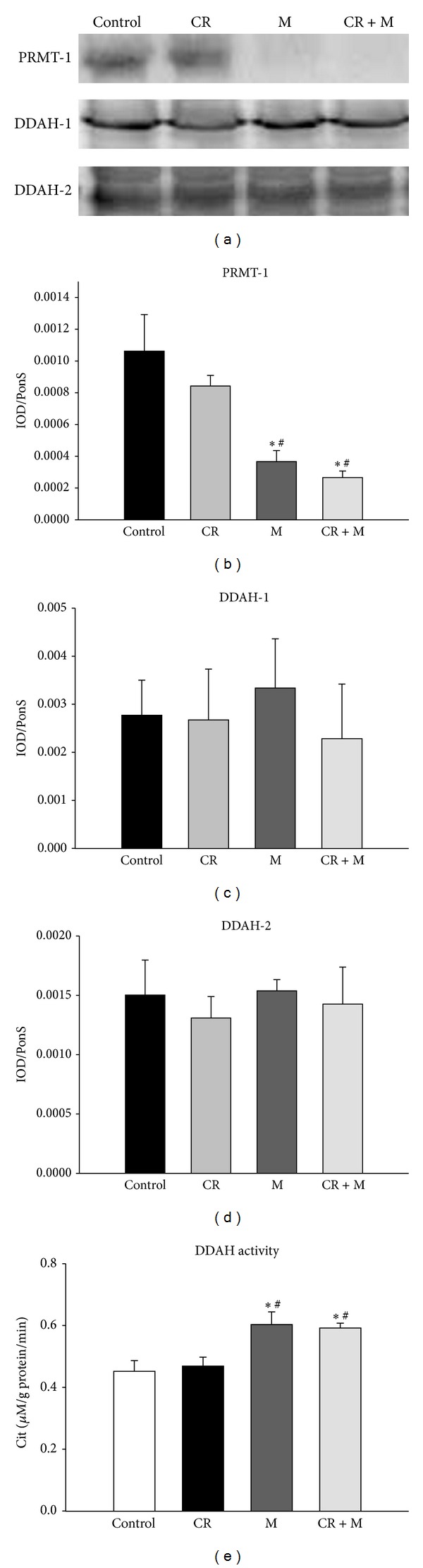
Representative western blots (a) showing protein arginine methyltransferase 1 (PRMT-1; ∼42 kDa), dimethylarginine dimethylaminohydrolase 1 (DDAH-1; ∼34 kDa), and DDAH-2 (∼30 kDa) bands in CR offspring at 12 weeks of age. Relative abundance of renal cortical (b) PRMT-1, (c) DDAH-1, and (d) DDAH-2. (e) Effect of melatonin and CR on renal DDAH activity in male offspring at 12 weeks of age. *P < 0.05 versus control; ^#^ *P* < 0.05 versus CR.

Next, we examined the expression/activity of proteins involved in the ADMA pathway. We found that renal level of protein arginine N-methyltransferase 1 (PRMT-1), an ADMA-synthesizing enzyme, was significantly lower in the M and CR + M groups than that in control and CR groups (Figure 2(b)). However, protein levels of DDAH-1 and -2, ADMA-metabolizing enzymes, in the kidney were not different between the four groups (Figures 2(c) and 2(d)). We found that renal DDAH activity did not differ between control and CR groups (Figure 2(e)). However, melatonin increased renal DDAH activity in both the M and CR + M groups. Thus, we speculate that the increase of systemic ADMA observed with CR is due to excessive synthesis or decreased metabolism in extrarenal tissues. On the other hand, the reduced plasma ADMA levels in response to melatonin might be due to decreased ADMA synthesis and increased ADMA breakdown in the kidney.

### 3.4. The Effects of Melatonin on Nephrogenesis

**Figure 3:**
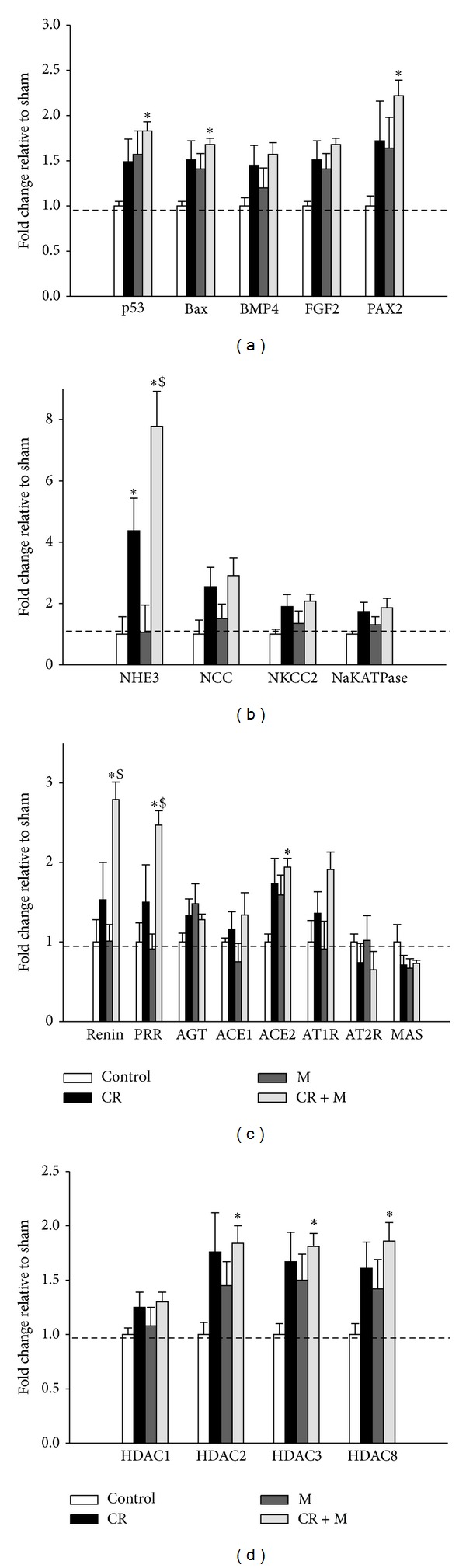
Effect of melatonin and CR on the expression of (a) apoptosis- and nephrogenesis-related genes, (b) sodium transporters, (c) renin-angiotensin system (RAS) components, and (d) class I histone deacetylase (HDAC) in the kidney at 12 weeks of age. *P < 0.05 versus control; ^#^ *P* < 0.05 versus CR; ^$^ *P* < 0.05 versus M.

We investigated whether changes in nephrogenesis- or apoptosis-related gene expression were associated with CR-induced reduced nephron numbers, as we found previously [10]. Consistent with our previous report [10], renal expression of p53 and the proapoptotic factor Bax did not differ between the control and CR groups (Figure 3(a)). Similarly, growth factors BMP4 and FGF2 were unaltered by CR or melatonin in the kidney. However, melatonin significantly increased the expression of the transcriptional activator PAX2 in CR + M group compared to controls (Figure 3(a)).

### 3.5. The Effects of Melatonin on Sodium Transporters, RAS, and HDACs

Next, we evaluated two critical pathways involved in hypertension, sodium transporters and RAS components. We found that CR upregulated sodium-hydrogen exchanger 3 (NHE3) expression in the kidney (Figure 3(b)). The increase in renal NHE3 expression was not prevented by melatonin therapy. CR had no effect on the expression of RAS genes in the kidney, including renin, PRR, AGT, ACE, ACE2, AT1R, AT2R, and MAS (Figure 3(c)). Melatonin treatment, on the other hand, upregulated renal expression of renin, PRR, and ACE2 in the CR + M group compared to the control. Because melatonin therapy prevented the elevation of BP in offspring exposed to maternal CR, our data suggested that the antihypertensive effect of melatonin was related to renin, PRR, and ACE2 expression in the CR model. We found that CR did not alter renal expression of class I HDACs in the CR versus control group (Figure 3(d)). However, melatonin therapy increased HDAC-2, -3, and -8 expression in the kidney.

**Figure 4:**
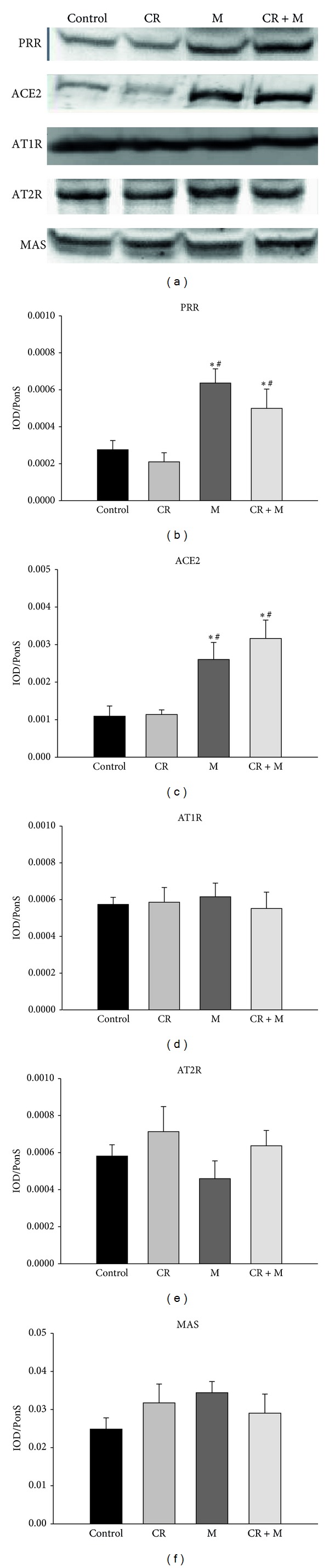
Representative western blots (a) showing prorenin receptor (PRR; 39 kDa), angiotensin-converting enzyme (ACE2; 50 kDa), angiotensin II type 1 (AT1R; 43 kDa) and type 2 (AT2R; 90 kDa), and MAS (37 kDa) proteins in male offspring kidneys at 12 weeks of age. Relative abundance of renal (b) PRR, (c) ACE2, (d) AT1R, (e) AT2R, and (f) MAS is quantified. *P < 0.05 versus control; ^#^ *P* < 0.05 versus CR.

We analyzed the renal protein levels of PRR, ACE2, AT1R, AT2R, and MAS. Melatonin therapy significantly increased renal PRR and ACE2 protein levels in the M and CR + M group compared with the control and CR groups (Figures 4(b) and 4(c)). We observed that renal AT1R, AT2R, and MAS protein levels did not differ among the four groups (Figures 4(d)–4(f)).

**Figure 5:**
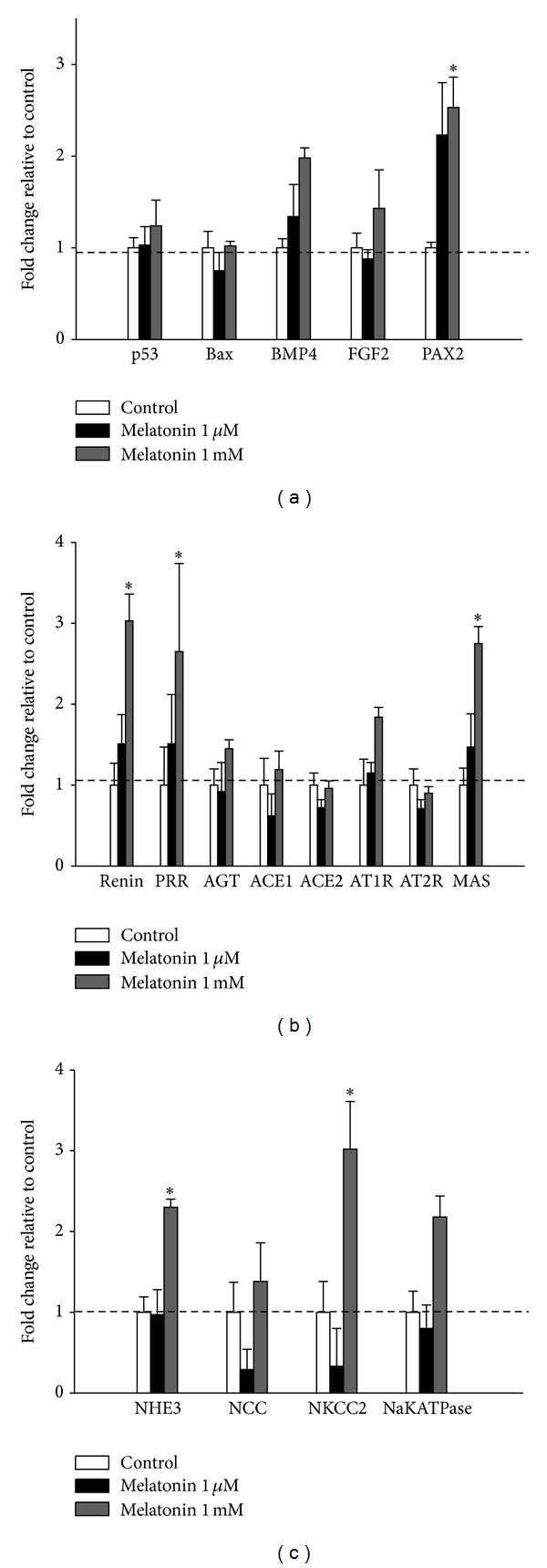
Gene expression of (a) apoptosis- and nephrogenesis-related genes, (b) RAS components, and (c) sodium transporters in the metanephroi of offspring from mothers treated with melatonin (1 *μ*M or 1 mM). *P < 0.05 versus control (*n* = 5/group).

We also determined whether melatonin regulated nephrogenesis-related genes, RAS components, sodium transporters, and HDACs during nephrogenesis. The mRNA levels in rat metanephroi grown in different concentrations of melatonin are shown in Figure 5. We found that low doses of melatonin had no effect on the expression of these genes, whereas high-dose melatonin treatment significantly increased expression of PAX2, renin, PRR, Mas, NHE3, and Na-K-Cl cotransporter 2 in metanephroi.

### 3.6. The Effects of Melatonin on Gene Expression during Nephrogenesis

**Figure 6:**
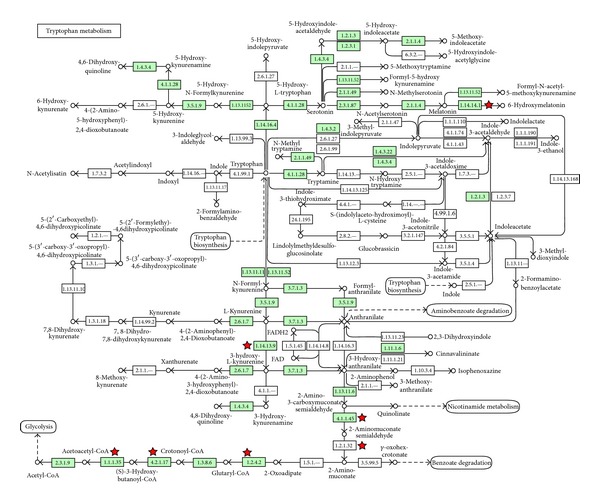
Enzymes of the tryptophan metabolism pathway that are regulated by melatonin therapy in the kidney (red stars). Data were analyzed using the KEGG Pathway feature of the DAVID software.

We demonstrated that numerous individual genes were significantly regulated in the kidneys of offspring from melatonin-treated mothers during a critical period of renal development. As shown in Table 4, 439 and 15 genes were upregulated and downregulated, respectively. The most significantly regulated biological theme in the KEGG gene ontology analysis was tryptophan metabolism (Figure 6).

## 4. Discussion

The major findings of our study can be summarized as follows: (1) CR offspring developed hypertension at 12 weeks of age and this was prevented by maternal melatonin therapy; (2) melatonin restored the CR-induced increase of plasma ADMA level, decreased l-arginine level, and decreased l-arginine-to-ADMA ratio; (3) CR reduced renal NO level and this was prevented by melatonin; (4) melatonin therapy increased PAX2 mRNA expression in the CR + M group; (5) CR upregulated renin and PRR expression and melatonin suppressed this increase; (6) melatonin therapy significantly increased renal ACE2 protein levels in the M and CR + M group; and (7) the expression of numerous genes was regulated in melatonin-treated offspring kidneys during nephrogenesis.

Our recent work indicates that ADMA-induced NO/reactive oxygen species (ROS) imbalance is involved in the development of hypertension in two different developmental models, maternal caloric restriction, and maternal diabetes [10, 11]. Several lines of evidence in this study indicated that ADMA-induced NO/ROS imbalance is involved in the developmental programming of hypertension in offspring exposed to maternal caloric restriction. First, plasma levels of the endogenous NOS inhibitor ADMA were increased in the CR group. Second, ADMA and l-arginine both compete for NOS and are present in a ratio that maintains NO homeostasis; this ratio was decreased in the plasma in the CR group. Third, maternal CR decreased renal NO levels in the offspring. Thus, alterations in the ADMA-NO pathway might be a major factor involved in programmed adult hypertension in response to maternal CR.

Melatonin is rapidly transferred from maternal to fetal circulation [20]. Administration of melatonin to pregnant rats prevents oxidative stress damage in the brains of offspring [21]. Previously, we showed that melatonin increases NO, restoring NO/ROS balance at the prehypertension stage and leading to lower blood pressure in young SHR [15]. Consistent with these findings, we found that early melatonin therapy in the mother could prevent programmed hypertension in their adult offspring. Thus, we suggest that melatonin has a novel protective effect on programmed hypertension through acting on the ADMA-NO pathway.

In addition to oxidative stress, the RAS plays a fundamental role in the development of hypertension and kidney development [5]. Epigenetic regulation of several RAS components has been reported in different programmed hypertension models [22, 23]. We demonstrated for the first time that melatonin therapy during nephrogenesis increased renin, PRR, and ACE2 expression in the kidney of the adult offspring. Consistent with these data, renal protein levels of PRR and ACE2 were increased in melatonin-treated offspring. Renin-PRR signaling is essential for proper kidney development and is causally linked to hypertension [13]. ACE2 appears to antagonize the effects of ACE through the production of angiotensin (1–7) in a manner that opposes the development of hypertension [24]. Surprisingly, melatonin therapy increased ACE2 expression in the kidney and prevented CR-induced programmed hypertension, despite the presence of increased renin and PRR expression. Notably, melatonin upregulated several RAS components and had reciprocal effects on vasodilation and vasoconstriction in rats at 3 months of age. Future studies are required to clarify the underlying mechanisms involved in the differential regulation of RAS components by melatonin.

Long-term amelioration of hypertension by melatonin therapy during gestation and lactation may be due to epigenetic changes in the kidney during a critical period of nephrogenesis. We found that melatonin upregulated HDAC-2, -3, and -8 expression in the kidney in CR + M group. This finding is consistent with that of our previous study showing that melatonin increased the expression of both class I and class II HDACs in vitro [25]. Given that melatonin increased class I HDACs expression and that HDACs are primarily thought to repress gene transcription, melatonin likely upregulates gene expression. Conversely, melatonin is known as a class III HDAC inhibitor [17]. Thus, melatonin might have dual effects on HDACs to epigenetically regulate gene expression. To the best of our knowledge, our study is the first to document altered expression of more than 400 genes in the kidney in response to melatonin and implicates melatonin in the protection from programmed hypertension in adult life. Notably, our data imply that melatonin is liable to induce, but not suppress, gene expression in the developing kidney. Using the KEGG database, several biological pathways were proposed to be regulated by melatonin including focal adhesion signaling, the peroxisome proliferator-activated receptors signaling pathway, fatty acid metabolism, the transforming growth factor *β* signaling pathway, and the Wnt signaling pathway. These findings suggest that melatonin might have a global epigenetic effect during nephrogenesis. Interestingly, the most significantly regulated biological theme was tryptophan metabolism, indicating that melatonin might have a negative feedback effect on its precursor tryptophan. Notably, maternal melatonin therapy has adverse effects on survival and renal growth in Wistar-Kyoto rats [26]. Because our data showed that maternal melatonin therapy had strong epigenetic effects, further evaluation is warranted to determine whether early melatonin therapy causes long-term epigenetic changes that lead to adverse effects in adulthood.

Previously, we showed that maternal CR reduces nephron numbers in offspring [10]. Increases in renal apoptosis and impaired expression of nephrogenesis-related genes may contribute to this reduction. In contrast to several earlier reports [27, 28], we found that apoptosis- and nephrogenesis-related genes were not altered in maternal CR-induced programmed hypertension. Of note, we showed for the first time that melatonin treatment upregulated PAX2 mRNA in metanephroi. Because PAX2 plays a crucial role in kidney development and is associated with various congenital renal and ureteral malformations, further studies are warranted to understand the epigenetic regulation of melatonin on PAX2 during nephrogenesis.

We conclude that prenatal melatonin therapy offsets the effects of maternal CR-induced programmed hypertension in adult offspring, primarily through the restoration of the ADMA-NO balance in the kidney. Our data suggested that a critical window exists during nephrogenesis in which the adult BP can be modified. Moreover, we showed that melatonin can modulate type I HDACs and serve as an inducer of gene expression in the developing kidney. The implications of melatonin-induced epigenetic changes on programmed hypertension in later life remain to be explored.

## Figures and Tables

**Table 1:** PCR primers sequences.

Gene	Forward	Reverse
Bax	5 ttgctgatggcaacttcaactg 3	5 ctttagtgcacagggccttgag 3
P53	5 catgagcgttgctctgatg 3	5 cagatactcagcatacggatttcc 3
PAX2	5 gagactcccagagtggtgtg 3	5 cattcccctgttctgatttg 3
FGF2	5 ccagttggtatgtggcactg 3	5 cagggaagggtttgacaaga 3
BMP4	5 gacttcgaggcgacacttctg 3	5 agccggtaaagatccctcatg 3
Renin	5 aacattaccagggcaactttcact 3	5 acccccttcatggtgatctg 3
Prorenin receptor	5 gaggcagtgaccctcaacat 3	5 ccctcctcacacaacaaggt 3
Angiotensinogen	5 gcccaggtcgcgatgat 3	5 tgtacaagatgctgagtgaggcaa 3
ACE	5 caccggcaaggtctgctt 3	5 cttggcatagtttcgtgaggaa 3
ACE2	5 acccttcttacatcagccctactg 3	5 tgtccaaaacctaccccacatat 3
AT1R	5 gctgggcaacgagtttgtct 3	5 cagtccttcagctggatcttca 3
AT2R	5 caatctggctgtggctgactt 3	5 tgcacatcacaggtccaaaga 3
MAS	5 catctctcctctcggctttgtg 3	5 cctcatccggaagcaaagg 3
HDAC-1	5 gaactggggacctacggg 3	5 gctcttgacaaattccacacac 3
HDAC-2	5 agttgcccttgattgtgaga 3	5 ccactgttgtccttggatttat 3
HDAC-3	5 tgatgaccagagttacaagcac 3	5 gggcaacatttc ggacag 3
HDAC-8	5 gctacccccggtttatatttacag 3	5 ttcgatcagagagtgaaccatactg 3
R18S	5 gccgcggtaattccagctcca 3	5 cccgcccgctcccaagatc 3

**Table 2:** Morphological and biochemical values in different experimental groups.

	Control	CR	M	CR + M
	*n* = 8	*n* = 8	*n* = 10	*n* = 8
Mortality	10%	0%	0%	0%
Body weight (BW) (g)	435 ± 14	356 ± 4*	489 ± 8^∗#^	370 ± 9^∗$^
Left kidney weight (g)	1.22 ± 0.06	1.01 ± 0.02	1.97 ± 0.05^∗#^	1.48 ± 0.03^#$^
Left kidney weight/100g BW	0.28 ± 0.01	0.28 ± 0.01	0.4 ± 0.01^∗#^	0.4 ± 0.01^∗#^
Heart weight (g)	1.23 ± 0.05	1.24 ± 0.02	1.63 ± 0.01^∗#^	1.16 ± 0.05^$^
Heart weight/100 g BW	0.28 ± 0.01	0.35 ± 0.01*	0.35 ± 0.01*	0.31 ± 0.01
Systolic blood pressure (mmHg)	162 ± 2	180 ± 2*	155 ± 1^#^	166 ± 1^$^
Diastolic blood pressure (mmHg)	122 ± 2	134 ± 3*	108 ± 2^∗#^	113 ± 1^∗#^
Mean arterial pressure (mmHg)	135 ± 2	149 ± 2*	124 ± 1^∗#^	131 ± 1^#$^
CCr, mL·min^−1^·kg body weight^−1^	9.12 ± 3.45	8.5 ± 3.0	7.34 ± 2.32	7.81 ± 2.76

CCr: clearance of creatinine; *P < 0.05 versus control; ^#^ *P* < 0.05 versus CR; ^$^ *P* < 0.05 versus M.

**Table 3:** L-Citrulline, L-arginine, and dimethylarginine levels in the plasma and kidney.

	Control	CR	M	CR + M
Plasma (*μ*mol)				
L-Citrulline	50 ± 4.1	61 ± 3.6	59.3 ± 5.1	55.8 ± 6.9
L-Arginine	121.1 ± 14	84.4 ± 2.4*	113.6 ± 8.7^#^	112.8 ± 13.6^#^
ADMA	1.31 ± 0.1	2.21 ± 0.18*	1.18 ± 0.06^#^	1.08 ± 0.12^#^
SDMA	0.66 ± 0.04	1.62 ± 0.27*	0.97 ± 0.09^#^	0.92 ± 0.08^#^
L-Arginine-to-ADMA ratio	92 ± 8	40 ± 4*	98 ± 10^#^	105 ± 6^#^
Kidney (*μ*mol/mg protein)				
L-Citrulline	52.5 ± 8.6	53.1 ± 4.6	97.6 ± 8.4	68.8 ± 12.4
L-Arginine	425 ± 62.3	552.9 ± 58.9	522.8 ± 61.6	488.1 ± 56
ADMA	5.09 ± 0.88	6.33 ± 0.71	6.72 ± 1.03	4.84 ± 0.61
SDMA	4.3 ± 0.65	5.3 ± 0.51	5.57 ± 0.79	4.59 ± 0.73
L-Arginine-to-ADMA ratio	86 ± 4	89 ± 5	80 ± 4	103 ± 8^$^

*P < 0.05 versus control; ^#^ *P* < 0.05 versus CR; ^$^ *P* < 0.05 versus M.

**Table 4:** Genes that changed by RPKM > 0.3 in the kidney of melatonin treated offspring versus control at 1 week of age.

Gene_ID	Gene symbol	Fold changes	Log_2_	*P* value
Upregulated: 439 genes				
ENSRNOG00000038989	D3ZSD6_RAT	28.686	4.842	0.0083
ENSRNOG00000006367	Slc5a8	19.264	4.268	0.0001
ENSRNOG00000003038	Sft2d2	17.101	4.096	0.0020
ENSRNOG00000007720	F1LX97_RAT	13.841	3.791	0.0027
ENSRNOG00000019014	Ndst1	12.657	3.662	0.0003
ENSRNOG00000017434	Mgat3	12.364	3.628	0.0005
ENSRNOG00000001656	Kcnj15	11.724	3.551	0.0028
ENSRNOG00000021292	—	11.449	3.517	0.0015
ENSRNOG00000017078	Sepn1	11.107	3.473	0.0011
ENSRNOG00000030121	Enpep	10.970	3.456	0.0029
ENSRNOG00000005854	Angpt1	10.920	3.449	0.0165
ENSRNOG00000009944	LOC314407	10.858	3.441	0.0017
ENSRNOG00000013279	Scd	10.775	3.430	0.0012
ENSRNOG00000001724	LOC678704	10.690	3.418	0.0011
ENSRNOG00000002463	LOC682752	10.633	3.411	0.0033
ENSRNOG00000011630	Ak3l1	10.304	3.365	0.0441
ENSRNOG00000005447	RGD1311564	10.117	3.339	0.0026
ENSRNOG00000009019	Slc6a6	10.090	3.335	0.0016
ENSRNOG00000002969	Itpkb	9.892	3.306	0.0020
ENSRNOG00000037307	Spata22	9.876	3.304	0.0036
ENSRNOG00000039717	Ipo11	9.584	3.261	0.0069
ENSRNOG00000025372	Glce	9.536	3.253	0.0023
ENSRNOG00000037884	Oxgr1	9.510	3.249	0.0169
ENSRNOG00000021203	Atl3	9.487	3.246	0.0056
ENSRNOG00000006787	Dhcr24	9.328	3.222	0.0023
ENSRNOG00000015038	Adam10	9.279	3.214	0.0005
ENSRNOG00000002519	Magt1	9.253	3.210	0.0010
ENSRNOG00000038933	D3ZF12_RAT	9.225	3.206	0.0023
ENSRNOG00000024757	RGD1310444	9.119	3.189	0.0066
ENSRNOG00000030285	Epha3	9.064	3.180	0.0032
ENSRNOG00000018338	Vwa1	9.017	3.173	0.0355
ENSRNOG00000022802	Tmem184b	8.982	3.167	0.0070
ENSRNOG00000013265	Tgfbr2	8.947	3.161	0.0014
ENSRNOG00000026941	Tril	8.934	3.159	0.0024
ENSRNOG00000020532	Kcnq1	8.904	3.154	0.0441
ENSRNOG00000018503	LOC293190	8.862	3.148	0.0172
ENSRNOG00000002198	LOC685352	8.711	3.123	0.0037
ENSRNOG00000017172	Fam125b	8.706	3.122	0.0291
ENSRNOG00000018554	—	8.663	3.115	0.0067
ENSRNOG00000013963	IL6RB_RAT	8.571	3.099	0.0023
ENSRNOG00000042565	—	8.547	3.095	0.0114
ENSRNOG00000032834	Hspa13	8.544	3.095	0.0011
ENSRNOG00000002355	Slc47a1	8.474	3.083	0.0025
ENSRNOG00000011927	SDC3_RAT	8.460	3.081	0.0065
ENSRNOG00000042540	Mef2a	8.454	3.080	0.0368
ENSRNOG00000029216	Dgcr2	8.331	3.059	0.0175
ENSRNOG00000023725	LOC689756	8.215	3.038	0.0191
ENSRNOG00000028129	Fktn	8.207	3.037	0.0063
ENSRNOG00000000547	Tspyl4	8.202	3.036	0.0103
ENSRNOG00000011859	Eif5a2	8.192	3.034	0.0403
ENSRNOG00000028387	E9PTK5_RAT	8.168	3.030	0.0197
ENSRNOG00000015986	Rassf8	8.134	3.024	0.0094
ENSRNOG00000029409	Gstm6l	8.062	3.011	0.0292
ENSRNOG00000008895	Hnf4a	7.993	2.999	0.0398
ENSRNOG00000038149	Defb9	7.948	2.991	0.0437
ENSRNOG00000040287	Cyp1b1	7.890	2.980	0.0439
ENSRNOG00000010468	Elovl6	7.871	2.977	0.0394
ENSRNOG00000014524	F1M9D3_RAT	7.828	2.969	0.0077
ENSRNOG00000014209	Utp6	7.793	2.962	0.0050
ENSRNOG00000013419	Agphd1	7.789	2.961	0.0031
ENSRNOG00000020653	S1pr2	7.775	2.959	0.0313
ENSRNOG00000018714	Arl5b	7.770	2.958	0.0078
ENSRNOG00000002408	Rbm47	7.719	2.948	0.0057
ENSRNOG00000008971	Hnf4g	7.715	2.948	0.0085
ENSRNOG00000011271	Mcc	7.688	2.943	0.0120
ENSRNOG00000002276	LOC100359714	7.641	2.934	0.0073
ENSRNOG00000009446	Rxra	7.607	2.927	0.0066
ENSRNOG00000019400	Dag1	7.591	2.924	0.0010
ENSRNOG00000013098	F1M9J1_RAT	7.581	2.922	0.0320
ENSRNOG00000014511	Alg10	7.551	2.917	0.0076
ENSRNOG00000012490	Amph	7.533	2.913	0.0461
ENSRNOG00000014934	Fam63b	7.481	2.903	0.0193
ENSRNOG00000039630	LOC290577	7.414	2.890	0.0045
ENSRNOG00000032707	Egf	7.368	2.881	0.0017
ENSRNOG00000015605	Ptprk	7.357	2.879	0.0298
ENSRNOG00000000168	Gatm	7.311	2.870	0.0017
ENSRNOG00000027097	F1M683_RAT	7.273	2.863	0.0087
ENSRNOG00000018109	Clic4	7.251	2.858	0.0048
ENSRNOG00000008629	Secisbp2l	7.236	2.855	0.0042
ENSRNOG00000019799	Pcdhgc3	7.231	2.854	0.0246
ENSRNOG00000024089	Fndc3b	7.221	2.852	0.0065
ENSRNOG00000015852	D4AD82_RAT	7.192	2.846	0.0020
ENSRNOG00000006967	Xiap	7.151	2.838	0.0136
ENSRNOG00000031487	F1LM52_RAT	7.129	2.834	0.0477
ENSRNOG00000014866	Pign	7.077	2.823	0.0190
ENSRNOG00000033206	Entpd5	7.060	2.820	0.0070
ENSRNOG00000037753	Slc10a2	7.002	2.808	0.0089
ENSRNOG00000040195	F1LZT0_RAT	7.001	2.808	0.0018
ENSRNOG00000042817	D4A5M8_RAT	6.925	2.792	0.0104
ENSRNOG00000005070	Spopl	6.920	2.791	0.0139
ENSRNOG00000006459	D4AEA4_RAT	6.871	2.781	0.0251
ENSRNOG00000012784	Gtf3c4	6.850	2.776	0.0096
ENSRNOG00000016968	Gramd4	6.838	2.774	0.0216
ENSRNOG00000004448	RGD1307051	6.819	2.770	0.0050
ENSRNOG00000021809	Gpx3	6.801	2.766	0.0008
ENSRNOG00000014183	Gnaq	6.801	2.766	0.0084
ENSRNOG00000012991	LOC100363275	6.798	2.765	0.0046
ENSRNOG00000013443	Tm9sf3	6.791	2.764	0.0040
ENSRNOG00000042673	LOC100359544	6.789	2.763	0.0012
ENSRNOG00000003873	Cpd	6.767	2.758	0.0028
ENSRNOG00000007990	Adipor2	6.762	2.758	0.0026
ENSRNOG00000007804	C1galt1	6.762	2.757	0.0109
ENSRNOG00000043256	D3ZNR8_RAT	6.720	2.749	0.0145
ENSRNOG00000015124	Gpam	6.720	2.748	0.0109
ENSRNOG00000004888	Spred2	6.690	2.742	0.0454
ENSRNOG00000003960	Tmem27	6.682	2.740	0.0026
ENSRNOG00000015750	Wnt7b	6.654	2.734	0.0218
ENSRNOG00000030763	Dpp4	6.601	2.723	0.0011
ENSRNOG00000039504	Q5M885_RAT	6.562	2.714	0.0116
ENSRNOG00000032768	D3Z9G8_RAT	6.497	2.700	0.0214
ENSRNOG00000039771	LOC100361629	6.494	2.699	0.0140
ENSRNOG00000009274	Fut11	6.475	2.695	0.0354
ENSRNOG00000027938	RGD1562037	6.420	2.683	0.0117
ENSRNOG00000001335	Zkscan1	6.419	2.682	0.0077
ENSRNOG00000004978	Prkacb	6.379	2.673	0.0216
ENSRNOG00000005446	Gna11	6.363	2.670	0.0172
ENSRNOG00000003884	Acmsd	6.362	2.669	0.0262
ENSRNOG00000028190	D4ACF8_RAT	6.354	2.668	0.0432
ENSRNOG00000006338	Lrp6	6.351	2.667	0.0041
ENSRNOG00000009523	Rab11fip2	6.345	2.666	0.0470
ENSRNOG00000003759	Galc	6.345	2.666	0.0140
ENSRNOG00000010620	NDC1_RAT	6.319	2.660	0.0275
ENSRNOG00000001821	Adipoq	6.306	2.657	0.0244
ENSRNOG00000038572	RGD1562646	6.293	2.654	0.0106
ENSRNOG00000026120	Fam8a1	6.282	2.651	0.0129
ENSRNOG00000025476	RGD1359349	6.243	2.642	0.0126
ENSRNOG00000019508	Wars2	6.216	2.636	0.0317
ENSRNOG00000008271	Fam91a1	6.216	2.636	0.0031
ENSRNOG00000017120	Abhd2	6.208	2.634	0.0278
ENSRNOG00000010843	Nhlrc3	6.203	2.633	0.0255
ENSRNOG00000030704	F1LV74_RAT	6.139	2.618	0.0369
ENSRNOG00000002509	Gnl3l	6.129	2.616	0.0124
ENSRNOG00000010841	Col8a2	6.089	2.606	0.0457
ENSRNOG00000002728	Btc	6.088	2.606	0.0348
ENSRNOG00000027320	Eif2c1	6.082	2.605	0.0243
ENSRNOG00000009453	Mobkl2b	6.072	2.602	0.0271
ENSRNOG00000007797	Rbpsuh	6.069	2.602	0.0133
ENSRNOG00000017286	HYES_RAT	6.064	2.600	0.0032
ENSRNOG00000002461	Nid1	6.057	2.599	0.0014
ENSRNOG00000006649	Thrb	6.048	2.596	0.0180
ENSRNOG00000025042	Pde3a	6.048	2.596	0.0189
ENSRNOG00000015916	Ttc38	6.048	2.596	0.0384
ENSRNOG00000013581	Extl3	6.038	2.594	0.0093
ENSRNOG00000002332	MSPD1_RAT	6.034	2.593	0.0213
ENSRNOG00000032757	D3Z903_RAT	6.032	2.593	0.0431
ENSRNOG00000029651	Rdh2	6.025	2.591	0.0258
ENSRNOG00000018588	Sox4	6.019	2.590	0.0342
ENSRNOG00000012428	Maf	6.005	2.586	0.0483
ENSRNOG00000009506	Mre11a	6.005	2.586	0.0332
ENSRNOG00000028330	—	5.987	2.582	0.0375
ENSRNOG00000034025	D4A4T5_RAT	5.979	2.580	0.0227
ENSRNOG00000007079	Met	5.979	2.580	0.0117
ENSRNOG00000008088	Btbd3	5.979	2.580	0.0181
ENSRNOG00000017546	Mylk3	5.945	2.572	0.0224
ENSRNOG00000042333	Dnal1	5.895	2.559	0.0187
ENSRNOG00000001092	Kl	5.873	2.554	0.0106
ENSRNOG00000016498	—	5.836	2.545	0.0049
ENSRNOG00000037765	Lims1	5.833	2.544	0.0367
ENSRNOG00000010267	Klhdc10	5.827	2.543	0.0346
ENSRNOG00000043277	D3ZIC7_RAT	5.809	2.538	0.0206
ENSRNOG00000024799	D3ZNV9_RAT	5.803	2.537	0.0032
ENSRNOG00000004919	Gns	5.795	2.535	0.0282
ENSRNOG00000015080	Wdfy1	5.766	2.528	0.0292
ENSRNOG00000009565	Pdk4	5.764	2.527	0.0206
ENSRNOG00000013082	LCAP_RAT	5.754	2.525	0.0322
ENSRNOG00000026501	Slc6a19	5.742	2.522	0.0406
ENSRNOG00000009597	Cyp4a1	5.740	2.521	0.0123
ENSRNOG00000011560	Mtmr9	5.738	2.521	0.0368
ENSRNOG00000022710	Prrg4	5.736	2.520	0.0269
ENSRNOG00000013469	LOC100362805	5.715	2.515	0.0059
ENSRNOG00000024640	RGD1304731	5.698	2.510	0.0080
ENSRNOG00000018952	Sema3g	5.692	2.509	0.0143
ENSRNOG00000020011	Q66HF5_RAT	5.677	2.505	0.0381
ENSRNOG00000012826	Creb3l2	5.665	2.502	0.0189
ENSRNOG00000032492	Usp22	5.657	2.500	0.0107
ENSRNOG00000021840	LOC500046	5.644	2.497	0.0118
ENSRNOG00000034026	Lclat1	5.642	2.496	0.0223
ENSRNOG00000009153	Cidec	5.642	2.496	0.0432
ENSRNOG00000028899	Zbtb33	5.633	2.494	0.0168
ENSRNOG00000001766	Tfrc	5.613	2.489	0.0102
ENSRNOG00000017901	Acy3	5.613	2.489	0.0044
ENSRNOG00000012095	Pkia	5.596	2.484	0.0339
ENSRNOG00000001796	Dgkg	5.573	2.479	0.0471
ENSRNOG00000004958	RGD1304605	5.563	2.476	0.0100
ENSRNOG00000025587	Plagl1	5.550	2.472	0.0289
ENSRNOG00000027540	Fam102b	5.536	2.469	0.0410
ENSRNOG00000001518	Itga6	5.519	2.465	0.0452
ENSRNOG00000032723	Eftud1	5.515	2.463	0.0336
ENSRNOG00000002053	F1M3H3_RAT	5.491	2.457	0.0081
ENSRNOG00000003472	Atp11c-ps1	5.473	2.452	0.0317
ENSRNOG00000003984	Apln	5.448	2.446	0.0337
ENSRNOG00000012453	RGD1564560	5.438	2.443	0.0046
ENSRNOG00000017846	Slc44a1	5.422	2.439	0.0293
ENSRNOG00000016921	Klhl11	5.418	2.438	0.0275
ENSRNOG00000026415	D4A301_RAT	5.403	2.434	0.0280
ENSRNOG00000013798	Fnbp1l	5.391	2.431	0.0098
ENSRNOG00000003620	Fmo3	5.384	2.429	0.0050
ENSRNOG00000018220	Pde4dip	5.377	2.427	0.0462
ENSRNOG00000000145	Pik3r3	5.352	2.420	0.0210
ENSRNOG00000008834	LOC306096	5.351	2.420	0.0356
ENSRNOG00000025882	Nipal1	5.345	2.418	0.0306
ENSRNOG00000010996	Mobkl1a	5.341	2.417	0.0147
ENSRNOG00000001582	Bach1	5.339	2.417	0.0199
ENSRNOG00000022309	D3ZRU8_RAT	5.313	2.410	0.0048
ENSRNOG00000015741	Slc2a13	5.298	2.406	0.0371
ENSRNOG00000014303	F1M753_RAT	5.294	2.404	0.0391
ENSRNOG00000036798	Dusp3	5.284	2.402	0.0199
ENSRNOG00000012142	Glyat	5.283	2.401	0.0081
ENSRNOG00000024426	D3ZXW1_RAT	5.259	2.395	0.0477
ENSRNOG00000006628	Dusp16	5.256	2.394	0.0271
ENSRNOG00000026143	Ckap2l	5.230	2.387	0.0271
ENSRNOG00000018867	Klhdc7a	5.223	2.385	0.0489
ENSRNOG00000025296	Lrrc8a	5.203	2.379	0.0176
ENSRNOG00000014508	Mgll	5.203	2.379	0.0137
ENSRNOG00000000589	RGD1310495	5.199	2.378	0.0372
ENSRNOG00000014234	Hif1an	5.192	2.376	0.0394
ENSRNOG00000008450	LOC100359539	5.178	2.372	0.0409
ENSRNOG00000010744	Nrp1	5.177	2.372	0.0072
ENSRNOG00000039837	RGD1563945	5.161	2.368	0.0466
ENSRNOG00000013177	Map3k1	5.154	2.366	0.0114
ENSRNOG00000021719	F1LX81_RAT	5.153	2.365	0.0133
ENSRNOG00000024629	Hadha	5.116	2.355	0.0126
ENSRNOG00000014907	Aldh8a1	5.105	2.352	0.0055
ENSRNOG00000036673	Sectm1b	5.098	2.350	0.0121
ENSRNOG00000024794	Senp5	5.096	2.349	0.0264
ENSRNOG00000005131	Lin7c	5.086	2.347	0.0289
ENSRNOG00000002225	Scarb2	5.081	2.345	0.0116
ENSRNOG00000020284	Prkar2a	5.077	2.344	0.0215
ENSRNOG00000014648	Efnb2	5.072	2.343	0.0303
ENSRNOG00000002488	Galnt10	5.063	2.340	0.0437
ENSRNOG00000017406	Atrnl1	5.056	2.338	0.0269
ENSRNOG00000010813	Tspan14	5.048	2.336	0.0304
ENSRNOG00000000645	Reep3	5.047	2.336	0.0262
ENSRNOG00000018873	Fam168a	5.036	2.332	0.0160
ENSRNOG00000020253	RAB1B_RAT	5.030	2.331	0.0128
ENSRNOG00000001235	Gna12	5.012	2.325	0.0149
ENSRNOG00000040215	F1LZL1_RAT	5.011	2.325	0.0302
ENSRNOG00000011619	Myo9a	4.988	2.319	0.0163
ENSRNOG00000039976	D3ZHG3_RAT	4.983	2.317	0.0137
ENSRNOG00000016011	Plekhg1	4.971	2.314	0.0315
ENSRNOG00000037909	Ppm1f	4.964	2.312	0.0269
ENSRNOG00000016419	Pdlim5	4.962	2.311	0.0248
ENSRNOG00000023280	Als2	4.952	2.308	0.0166
ENSRNOG00000005417	Zhx2	4.948	2.307	0.0430
ENSRNOG00000017671	Rasa3	4.944	2.306	0.0403
ENSRNOG00000016848	Fzd4	4.942	2.305	0.0255
ENSRNOG00000003508	LOC100364400	4.942	2.305	0.0244
ENSRNOG00000012394	Bcl2l13	4.931	2.302	0.0466
ENSRNOG00000018400	D4AEL2_RAT	4.931	2.302	0.0303
ENSRNOG00000013707	Spata13	4.930	2.302	0.0445
ENSRNOG00000002039	LOC100360066	4.930	2.301	0.0436
ENSRNOG00000004563	Sec24a	4.917	2.298	0.0191
ENSRNOG00000020386	D3ZKH4_RAT	4.906	2.295	0.0098
ENSRNOG00000007419	Pank3	4.900	2.293	0.0128
ENSRNOG00000024533	Aer61	4.889	2.290	0.0382
ENSRNOG00000027151	Lrrc58	4.886	2.289	0.0393
ENSRNOG00000030124	Ptpn11	4.869	2.284	0.0160
ENSRNOG00000006131	Mettl2	4.846	2.277	0.0271
ENSRNOG00000000407	Dcbld1	4.834	2.273	0.0412
ENSRNOG00000008061	Nuak1	4.826	2.271	0.0360
ENSRNOG00000037514	Qser1	4.821	2.269	0.0136
ENSRNOG00000004959	Actr2	4.807	2.265	0.0327
ENSRNOG00000028582	F1M163_RAT	4.795	2.261	0.0045
ENSRNOG00000043037	LOC100366023	4.788	2.259	0.0349
ENSRNOG00000012135	F1M2H7_RAT	4.763	2.252	0.0406
ENSRNOG00000031069	D4A9A7_RAT	4.749	2.247	0.0462
ENSRNOG00000023109	F1LVL2_RAT	4.736	2.244	0.0482
ENSRNOG00000004442	RGD1311756	4.729	2.241	0.0456
ENSRNOG00000021318	Epas1	4.723	2.240	0.0138
ENSRNOG00000018099	Itch	4.702	2.233	0.0383
ENSRNOG00000038892	LOC686123	4.691	2.230	0.0268
ENSRNOG00000000296	Aqp6	4.685	2.228	0.0310
ENSRNOG00000014901	Uggt1	4.684	2.228	0.0168
ENSRNOG00000019659	Aspa	4.680	2.227	0.0055
ENSRNOG00000010450	D4ADY9_RAT	4.662	2.221	0.0220
ENSRNOG00000011066	6-Mar	4.658	2.220	0.0264
ENSRNOG00000013121	Mier3	4.647	2.216	0.0408
ENSRNOG00000030894	Slco1a6	4.640	2.214	0.0068
ENSRNOG00000004964	Erbb3	4.609	2.205	0.0351
ENSRNOG00000014135	Rab11fip4	4.607	2.204	0.0453
ENSRNOG00000005052	Slc39a9	4.594	2.200	0.0454
ENSRNOG00000005276	Csnk2a1	4.589	2.198	0.0259
ENSRNOG00000015007	RGD1565591	4.583	2.196	0.0462
ENSRNOG00000002099	Wdfy3	4.579	2.195	0.0217
ENSRNOG00000001747	Pak2	4.572	2.193	0.0178
ENSRNOG00000018226	Zcchc14	4.565	2.190	0.0441
ENSRNOG00000010702	Ube3c	4.564	2.190	0.0154
ENSRNOG00000010610	Hpgd	4.556	2.188	0.0125
ENSRNOG00000001756	D3ZDR3_RAT	4.551	2.186	0.0486
ENSRNOG00000006335	Klhl9	4.550	2.186	0.0083
ENSRNOG00000016715	Kif11	4.547	2.185	0.0159
ENSRNOG00000021916	Slc16a12	4.541	2.183	0.0224
ENSRNOG00000011250	Inmt	4.506	2.172	0.0125
ENSRNOG00000013140	Pdzd2	4.502	2.171	0.0305
ENSRNOG00000012440	Msra	4.501	2.170	0.0308
ENSRNOG00000019932	Ip6k1	4.500	2.170	0.0307
ENSRNOG00000037227	Yes1	4.499	2.170	0.0412
ENSRNOG00000012054	Zmpste24	4.498	2.169	0.0179
ENSRNOG00000007370	Rnf144a	4.493	2.168	0.0443
ENSRNOG00000022968	F1M4Y9_RAT	4.491	2.167	0.0400
ENSRNOG00000011340	D3ZMJ4_RAT	4.488	2.166	0.0143
ENSRNOG00000021705	D3ZXN6_RAT	4.486	2.165	0.0229
ENSRNOG00000003865	Tmigd1	4.483	2.164	0.0072
ENSRNOG00000012105	F1MAE3_RAT	4.478	2.163	0.0346
ENSRNOG00000011312	F1LQ39_RAT	4.475	2.162	0.0366
ENSRNOG00000000127	F1LT58_RAT	4.463	2.158	0.0484
ENSRNOG00000022929	MTMRC_RAT	4.438	2.150	0.0307
ENSRNOG00000033372	Klhl24	4.431	2.148	0.0197
ENSRNOG00000008332	Smo	4.420	2.144	0.0209
ENSRNOG00000028616	Pck1	4.418	2.143	0.0219
ENSRNOG00000013281	Mib1	4.415	2.142	0.0306
ENSRNOG00000011448	Eri1	4.410	2.141	0.0414
ENSRNOG00000028422	Rmnd5a	4.409	2.141	0.0212
ENSRNOG00000014859	Rnf152	4.404	2.139	0.0298
ENSRNOG00000001893	LOC100362453	4.397	2.137	0.0349
ENSRNOG00000018123	Ccny	4.396	2.136	0.0173
ENSRNOG00000016337	Slc22a1	4.394	2.135	0.0356
ENSRNOG00000003709	Kmo	4.389	2.134	0.0166
ENSRNOG00000019939	CCND2_RAT	4.386	2.133	0.0383
ENSRNOG00000029947	—	4.377	2.130	0.0399
ENSRNOG00000008346	Itgb6	4.372	2.128	0.0245
ENSRNOG00000008678	Antxr1	4.357	2.123	0.0237
ENSRNOG00000029924	Klk1l	4.344	2.119	0.0267
ENSRNOG00000043406	LOC100360800	4.341	2.118	0.0323
ENSRNOG00000012343	Pdp2	4.324	2.112	0.0419
ENSRNOG00000009899	D3ZWL1_RAT	4.306	2.106	0.0427
ENSRNOG00000003434	Trove2	4.301	2.105	0.0368
ENSRNOG00000015519	Ces1d	4.294	2.102	0.0253
ENSRNOG00000017439	Cgnl1	4.294	2.102	0.0236
ENSRNOG00000014700	Ttc36	4.287	2.100	0.0266
ENSRNOG00000007944	Edem1	4.281	2.098	0.0367
ENSRNOG00000031263	Haao	4.246	2.086	0.0200
ENSRNOG00000001647	Ets2	4.245	2.086	0.0357
ENSRNOG00000008652	RGD1564964	4.226	2.079	0.0153
ENSRNOG00000023202	Usp15	4.217	2.076	0.0230
ENSRNOG00000016289	Bmpr1b	4.212	2.075	0.0370
ENSRNOG00000015024	E9PT54_RAT	4.208	2.073	0.0252
ENSRNOG00000000555	Eif4ebp2	4.199	2.070	0.0381
ENSRNOG00000008620	Smad3	4.198	2.070	0.0440
ENSRNOG00000008619	Agtrap	4.198	2.070	0.0217
ENSRNOG00000009711	Hepacam2	4.196	2.069	0.0409
ENSRNOG00000015734	Ube3a	4.193	2.068	0.0225
ENSRNOG00000015634	SMAD4_RAT	4.189	2.067	0.0277
ENSRNOG00000042519	RGD1312026	4.182	2.064	0.0380
ENSRNOG00000007564	Evc	4.160	2.057	0.0289
ENSRNOG00000008372	Vamp7	4.160	2.057	0.0433
ENSRNOG00000024671	D4AA13_RAT	4.157	2.056	0.0120
ENSRNOG00000004622	Calcrl	4.142	2.050	0.0131
ENSRNOG00000009660	Enpp6	4.140	2.050	0.0247
ENSRNOG00000014750	D3ZXU7_RAT	4.138	2.049	0.0176
ENSRNOG00000008694	Miox	4.134	2.048	0.0226
ENSRNOG00000004831	Arid2	4.134	2.047	0.0317
ENSRNOG00000043167	Itga9	4.124	2.044	0.0349
ENSRNOG00000001770	Ehhadh	4.114	2.040	0.0104
ENSRNOG00000042160	Tmem167b	4.112	2.040	0.0466
ENSRNOG00000018668	Glg1	4.095	2.034	0.0172
ENSRNOG00000007985	D4ABH6_RAT	4.084	2.030	0.0231
ENSRNOG00000014623	F1M3F2_RAT	4.071	2.026	0.0332
ENSRNOG00000002227	Kit	4.056	2.020	0.0429
ENSRNOG00000016219	Vnn1	4.052	2.019	0.0115
ENSRNOG00000008322	E9PTI4_RAT	4.035	2.013	0.0418
ENSRNOG00000011358	Hipk3	4.034	2.012	0.0372
ENSRNOG00000028335	Fat4	4.017	2.006	0.0190
ENSRNOG00000025554	Zfp445	4.009	2.003	0.0365
ENSRNOG00000003388	Cenpf	3.990	1.996	0.0146
ENSRNOG00000000614	Bicc1	3.987	1.995	0.0162
ENSRNOG00000039091	D3ZRC4_RAT	3.975	1.991	0.0145
ENSRNOG00000030154	Cyp4a2	3.962	1.986	0.0422
ENSRNOG00000033172	—	3.952	1.983	0.0176
ENSRNOG00000017466	Kif5b	3.949	1.981	0.0128
ENSRNOG00000042879	D4A3X0_RAT	3.943	1.979	0.0454
ENSRNOG00000002146	Pkd2	3.942	1.979	0.0358
ENSRNOG00000012940	Vps41	3.937	1.977	0.0280
ENSRNOG00000017291	Sord	3.928	1.974	0.0133
ENSRNOG00000001606	Adamts5	3.923	1.972	0.0420
ENSRNOG00000016534	D3ZKX0_RAT	3.904	1.965	0.0295
ENSRNOG00000007202	Sema3d	3.898	1.963	0.0254
ENSRNOG00000012436	Adh6	3.897	1.962	0.0137
ENSRNOG00000016334	Rod1	3.867	1.951	0.0167
ENSRNOG00000018011	RGD1564456	3.867	1.951	0.0417
ENSRNOG00000039494	D4A608_RAT	3.853	1.946	0.0328
ENSRNOG00000014976	Acsm2	3.850	1.945	0.0330
ENSRNOG00000006636	Otud6b	3.817	1.932	0.0439
ENSRNOG00000015849	Sepp1	3.812	1.930	0.0292
ENSRNOG00000004689	Ptdss1	3.811	1.930	0.0342
ENSRNOG00000013808	Ces2g	3.803	1.927	0.0345
ENSRNOG00000014673	Eri2	3.791	1.922	0.0429
ENSRNOG00000009819	Vezf1	3.784	1.920	0.0450
ENSRNOG00000016758	Loxl2	3.783	1.919	0.0310
ENSRNOG00000010061	Gmfb	3.763	1.912	0.0466
ENSRNOG00000023021	Msl2	3.746	1.906	0.0486
ENSRNOG00000039571	Glod5	3.742	1.904	0.0370
ENSRNOG00000017600	Ptpn9	3.739	1.903	0.0329
ENSRNOG00000000590	Naglt1	3.718	1.895	0.0365
ENSRNOG00000011511	Stk24	3.716	1.894	0.0398
ENSRNOG00000018279	Sfxn1	3.712	1.892	0.0213
ENSRNOG00000003953	RB3GP_RAT	3.709	1.891	0.0486
ENSRNOG00000024632	Atf6	3.699	1.887	0.0421
ENSRNOG00000016779	Fam120a	3.679	1.879	0.0257
ENSRNOG00000010379	Cugbp1	3.670	1.876	0.0295
ENSRNOG00000010780	Dlc1	3.664	1.874	0.0495
ENSRNOG00000003948	Llgl1	3.659	1.871	0.0486
ENSRNOG00000016183	Ipp	3.647	1.867	0.0466
ENSRNOG00000017964	Slc22a25	3.567	1.835	0.0201
ENSRNOG00000039745	Pm20d1	3.557	1.831	0.0300
ENSRNOG00000010107	PALLD_RAT	3.528	1.819	0.0467
ENSRNOG00000019444	D4ADJ6_RAT	3.515	1.814	0.0265
ENSRNOG00000011260	Cmbl	3.513	1.813	0.0221
ENSRNOG00000013322	DPOLA_RAT	3.505	1.810	0.0498
ENSRNOG00000039278	Mcart1	3.477	1.798	0.0345
ENSRNOG00000021108	Slc22a12	3.449	1.786	0.0263
ENSRNOG00000010887	RGD1309534	3.435	1.781	0.0382
ENSRNOG00000008331	RGD1309995	3.394	1.763	0.0425
ENSRNOG00000007949	Rgn	3.355	1.746	0.0276
ENSRNOG00000011987	Cd2ap	3.343	1.741	0.0306
ENSRNOG00000042175	B6VQA7_RAT	3.331	1.736	0.0384
ENSRNOG00000012190	Cldn2	3.324	1.733	0.0347
ENSRNOG00000023972	F1M6Q3_RAT	3.323	1.733	0.0322
ENSRNOG00000011763	Serp1	3.319	1.731	0.0316
ENSRNOG00000004496	Rock2	3.318	1.730	0.0337
ENSRNOG00000004677	Zeb2	3.306	1.725	0.0306
ENSRNOG00000013409	Gclm	3.301	1.723	0.0338
ENSRNOG00000004302	Pah	3.270	1.709	0.0368
ENSRNOG00000010947	MMP14_RAT	3.253	1.702	0.0337
ENSRNOG00000011058	Utrn	3.247	1.699	0.0378
ENSRNOG00000018215	Slc22a6	3.246	1.698	0.0377
ENSRNOG00000016456	Il33	3.234	1.693	0.0319
ENSRNOG00000002541	Pds5a	3.164	1.662	0.0449
ENSRNOG00000002680	Lamc1	3.139	1.650	0.0366
ENSRNOG00000011124	Eif4g2-ps1	3.125	1.644	0.0380
ENSRNOG00000004009	Xpnpep2	3.118	1.641	0.0414
ENSRNOG00000010768	Kpna4	3.114	1.639	0.0499
ENSRNOG00000042249	F1LTA7_RAT	3.101	1.633	0.0422
ENSRNOG00000014166	Smoc2	3.078	1.622	0.0413
ENSRNOG00000002305	Slc15a2	3.061	1.614	0.0400
ENSRNOG00000005130	Ogdh	3.049	1.608	0.0465
ENSRNOG00000018086	Slc22a8	3.048	1.608	0.0418
ENSRNOG00000010814	Bmpr1a	3.006	1.588	0.0432
ENSRNOG00000032885	CYC_RAT	2.936	1.554	0.0478

Down-regulated Genes: 15				
ENSRNOG00000032087	F1LWC2_RAT	0.301	−1.734	0.0328
ENSRNOG00000032609	—	0.300	−1.738	0.0394
ENSRNOG00000033748	F1LWC2_RAT	0.299	−1.741	0.0381
ENSRNOG00000025670	Shisa3	0.295	−1.759	0.0282
ENSRNOG00000029115	—	0.284	−1.815	0.0315
ENSRNOG00000011821	S100a4	0.228	−2.134	0.0086
ENSRNOG00000007632	Zmynd17	0.211	−2.243	0.0365
ENSRNOG00000025408	D3ZTT0_RAT	0.189	−2.403	0.0422
ENSRNOG00000028844	Slc9a5	0.181	−2.466	0.0282
ENSRNOG00000006889	Ambp	0.150	−2.741	0.0177
ENSRNOG00000028730	D3ZI71_RAT	0.148	−2.757	0.0450
ENSRNOG00000026067	Wfdc10	0.144	−2.791	0.0123
ENSRNOG00000037374	D3ZPQ1_RAT	0.140	−2.840	0.0087
ENSRNOG00000033517	LOC100360791	0.129	−2.950	0.0009
ENSRNOG00000042909	F1LZX4_RAT	0.107	−3.229	0.0466
ENSRNOG00000014578	Fxyd4	0.096	−3.374	0.0001
